# Effects of wearing a surgical face mask on cardiac biomarkers, respiratory function, and perceptual responses during exercise in a hot and humid climate at different intensities: a randomized crossover trial

**DOI:** 10.1186/s13102-026-01532-z

**Published:** 2026-02-03

**Authors:** Chen Zheng, Eric Tsz-Chun Poon, Jing-Lin Huang, Ke-Wen Wan, Feng-Hua Sun, Cindy Hui-Ping Sit, Martin Chi-Sang Wong, Jun-Hao Huang, Stephen Heung-Sang Wong

**Affiliations:** 1 Department of Health and Physical Education, Faculty of Liberal Arts and Social Sciences, The Education University of Hong Kong, Hong Kong, China; 2 Department of Sports Science and Physical Education, Faculty of Education, The Chinese University of Hong Kong, Hong Kong, China; 3 Guangdong Provincial Key Laboratory of Physical Activity and Health Promotion, Scientific Research Center, Guangzhou Sport University, Guangzhou, China; 4 Jockey Club School of Public Health and Primary Care, Faculty of Medicine, The Chinese University of Hong Kong, Hong Kong, China

**Keywords:** Cardio-related biomarker, Exercise intensity, Face mask, Heat, Respiratory function

## Abstract

**Purpose:**

Ongoing concerns have been raised about wearing face masks during exercise, yet large-scale experimental studies, particularly conducted under heat, are lacking. We aimed to investigate the effects of wearing a surgical face mask during exercise on cardiac biomarkers, respiratory function, and perceptual responses in a hot and humid condition at various intensities.

**Methods:**

A total of 98 participants (mean age, 20.4 years; 29 women) completed six separate experimental trials in a randomized order that consisted of exercising on a treadmill at light, moderate, or vigorous intensity with or without a surgical face mask (each trial, 15 min), in a climatic chamber set at 30 ± 1 ℃ and 70 ± 3% humidity. Cardiorespiratory and perceptual responses were measured pre- and post-exercise, continuously during exercise, and post-exercise only.

**Results:**

Cardiac biomarkers including creatine kinase myocardial band (CK-MB), N-terminal pro-brain natriuretic peptide, and troponin T values were higher at post-exercise, with CK-MB higher following high-intensity than light-intensity exercise, despite no significant effects of mask use. Similarly, blood pressure, lactate, and flow-mediated dilation levels, as well as mean value of forehead temperature, core-temperature, heart rate, and respiratory exchange ratio showed no significant difference with or without surgical face mask, although these values were generally higher at higher intensities and/or significantly increased post-exercise. Most respiratory parameters and perceived discomfort levels tended to show negative effects post-exercise with a surgical face mask under this condition.

**Conclusion:**

Wearing a surgical face mask for 15 min during exercise in hot and humid conditions had limited effects on cardio-related parameters but significantly affected respiratory function and increased discomfort, particularly at high intensities.

**Trial registration:**

This study was registered in the Chinese Clinical Trial Registry (#ChiCTR2100053144).

**Supplementary Information:**

The online version contains supplementary material available at 10.1186/s13102-026-01532-z.

## Background

In March 2020, the World Health Organization (WHO) declared coronavirus disease-2019 (COVID-19) a pandemic, with > 775 million confirmed cases and 7 million confirmed deaths as of June 24, 2024 [1]. Face masks were worn as a strategy to prevent COVID-19 spread through reducing aerosol and respiratory droplets [2]. While face-mask wearing prevalence varied significantly according to region, policy stringency, and time, many studies documented high prevalence rates in public settings across various countries [3, 4]. Maintaining regular exercise/physical activity (PA) is beneficial for physical and mental health, and was also beneficial for patients with COVID-19 during the pandemic [5, 6]. However, non-surgical face masks may exhibit leakage, reducing their effectiveness, particularly during exercise when increased breathing frequency and volume exacerbate airflow escape [7]. Despite the WHO's recommendation against wearing a face mask during vigorous exercise owing to the risk of reducing breathing capacity [8], a global survey of athletes reported that 70.8% wore face masks while exercising during the pandemic [9].

In this context, safety in relation to wearing a face mask during exercise has remained an on-going public concern. Inconsistent findings have been reported regarding the effects of wearing a face mask. Some studies have reported no effect on exercise performance [10], time to exhaustion [11], or maximal oxygen consumption (VO_2max_) [12], while others have reported a reduction in exercise duration and VO_2max_ [13, 14]. Our group recently conducted a systematic review of 45 relevant studies to investigate the effects of wearing different types of face masks during exercise on various physiological and psychological outcomes in healthy individuals [15]. The findings indicated that wearing face masks during exercise modestly affected both physiological and psychological parameters. Specifically, there was a reduction in oxygen uptake (standardized mean difference [SMD] − 0.66), a decrease in end-tidal partial pressure of oxygen (mean difference [MD] − 3.79 mmHg), and a decrease in carbon dioxide production (SMD − 0.77), along with an increase in end-tidal partial pressure of carbon dioxide (MD 2.93 mmHg). Additionally, participants reported significantly higher levels of dyspnea (SMD 0.72), fatigue (MD 1.34), and thermal sensation (SMD 0.67), although the overall effect on exercise performance appeared to be small [15]. However, some of the reviewed studies may have had potential carryover effects owing to insufficient washout periods between experimental and control trials, contributing to a risk of bias. In addition, difficulty breathing when wearing a face mask has been reported, even at rest, as the face mask increases breathing resistance, which may intensify over time as mist or water droplets condense inside the mask [16]. Despite these general findings, most of these studies employed incremental exercise trials to elicit maximal responses, with less data being available for steady-state prolonged exercise. Extrapolating intensity-specific effects of face mask use from incremental trials poses challenges, as accumulating fatigue across increasing intensities may confound results. Therefore, conducting separate trials at distinct exercise intensities is essential to accurately evaluate the intensity-specific effects of wearing face masks [15].

Consideration should also be given to the effects of temperature and humidity when wearing a face mask during exercise [17]. This is of particular relevance to regions with tropical or sub-tropical climates (such as Hong Kong, with humidity > 70% and temperature > 20 ℃ in most months). Under these hot and humid conditions, breathing frequency tends to increase during exercise [18]. Previous studies have also shown that face masks elevate the local skin micro-climate temperature around the mouth and perceived thermal discomfort [19, 20]; however, the core temperature remains unaffected during low-to-moderate and high-intensity exercise when compared with no-mask conditions [21, 22]. The combined effect of thermal discomfort and mask-induced discomfort, such as breathing resistance and facial irritation, may further elevate the perceptual strain associated with wearing a face mask [23]. Despite this, few experimental studies with relatively small sample sizes (*n* ≤ 12) have specifically examined the effect of face-mask use during exercise in hot conditions [19, 22, 24], and the use of incremental exercise protocols may confound intensity-specific responses owing to accumulating fatigue as aforementioned. Further research is needed to investigate the effect of wearing a face mask at distinct exercise intensities in a hot controlled environment. This would help provide precise, intensity-specific data to inform evidence-based exercise recommendations for populations in tropical or subtropical regions.

Furthermore, face mask-induced hypercapnia and dyspnea from carbon dioxide rebreathing may increase respiratory strain and discomfort during exercise [25], particularly in hot and humid conditions. This poses potential safety concerns, particularly for young adults who often engage in high-intensity activities. While previous studies have primarily focused on simpler biomarkers such as lactate and blood gas levels [26, 27], investigating the effects on cardiac-specific biomarkers [28], such as N-terminal pro-brain natriuretic peptide (NT-proBNP), troponin T (TNNT1), and creatine kinase-MB (CK-MB), alongside flow-mediated dilation (FMD) as an index of endothelial function, is warranted to better understand potential cardiovascular stress during exercise wearing masks in such environments. Therefore, this study aimed to investigate the effects of face-mask use on cardiac biomarkers, respiratory function, and perceptual responses to help inform safe exercise practices in hot and humid conditions. We hypothesized that wearing a surgical face mask would alter cardiac biomarkers, cardiovascular responses, respiratory function impairment, and perceived discomfort compared with no mask, with greater effects at higher exercise intensities in a hot and humid environment.

## Methods

### Participants

**Fig. 1:**
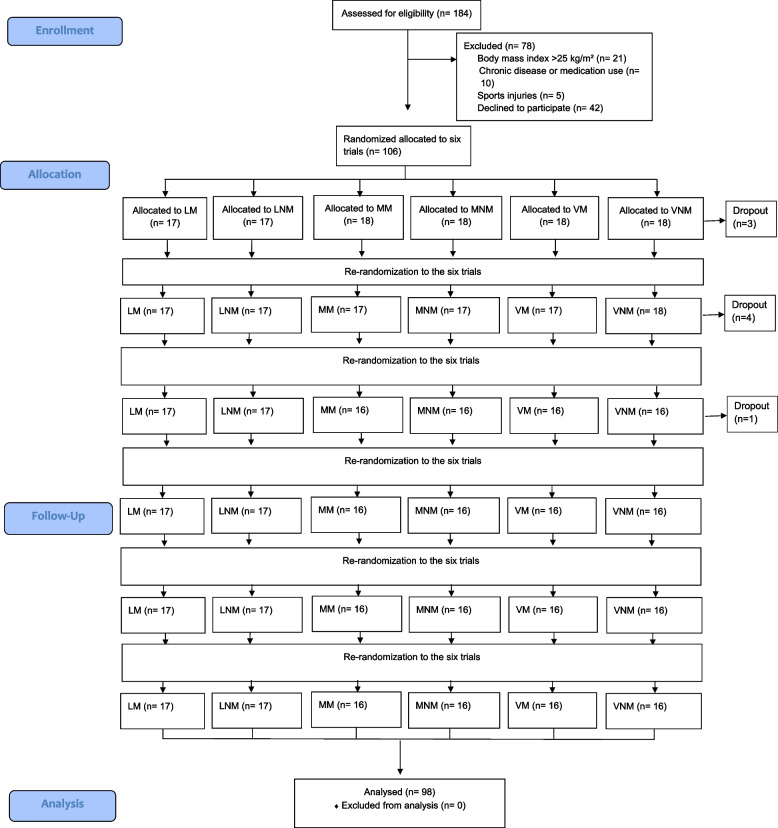
CONSORT flow diagram

Participants were recruited through advertisements at universities and partner institutions. Individuals who met the following inclusion criteria were invited to participate in this study: (i) Chinese young adults aged 18–30 years; (ii) those of normal weight (body mass index [BMI] ≤ 23.0 kg/m^2^) based on Asian criteria; (iii) those without any injury that might potentially influence exercise; and (iv) those without chronic disease or current medication. The sample size calculation was based on a previous study that investigated whether troponin T type 1 (TNNT1), a primary cardiac biomarker, would increase following an acute bout of exercise [29]. A total of 90 participants were required to determine an anticipated effect size (0.2) using a randomized crossover design with a two-sided significance level of 0.05% and 80% power. Based on previous experience, we calculated a potential withdrawal rate of 10% and aimed to recruit 100 study participants (Fig. 1). After receiving a detailed explanation of the study, all the participants signed an informed consent form. This study was approved by the Joint Chinese University of Hong Kong-New Territories East Cluster Clinical Research Ethics Committee (CREC Ref. No.:2021.546) and was registered at the Chinese Clinical Trial Registry (ChiCTR2100053144). The study was conducted in accordance with the Declaration of Helsinki.

### Study design

**Fig. 2:**
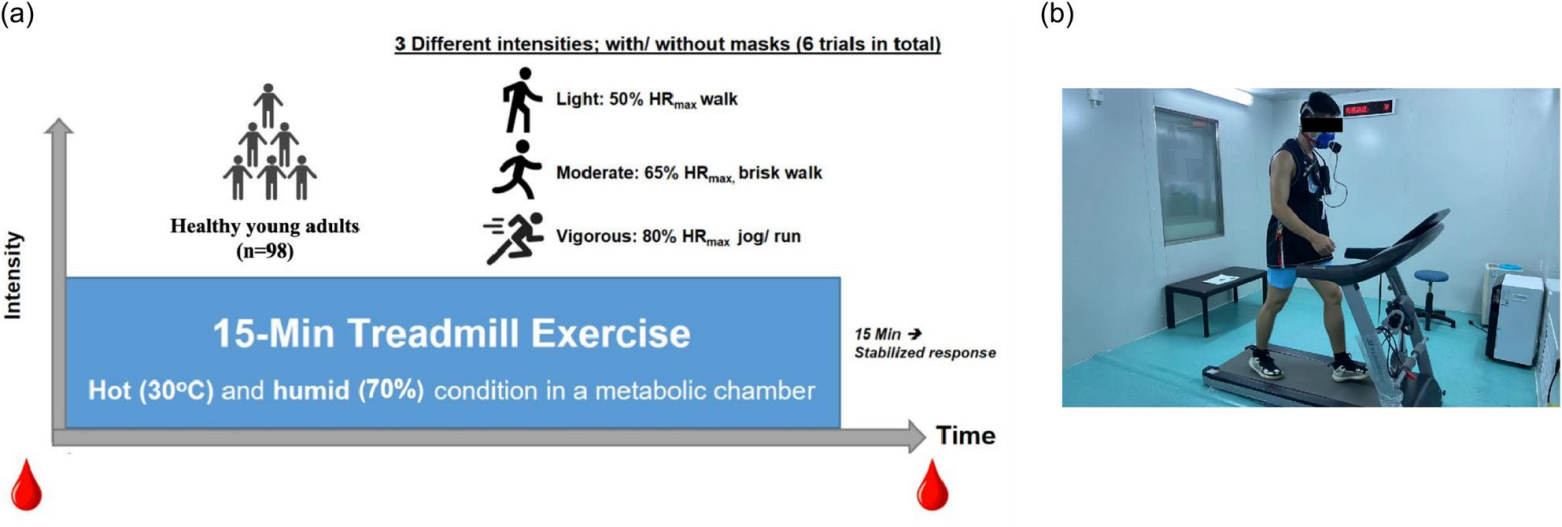
Exercise protocol (**a**) study design; (**b**) illustration of exercise in a hot and humid chamber

This randomized crossover study involved six experimental trials, namely, light-intensity exercise wearing a surgical face mask (LM), light-intensity exercise not wearing a surgical face mask (LNM), moderate-intensity exercise wearing a surgical face mask (MM), moderate-intensity exercise not wearing a surgical face mask (MNM), vigorous-intensity exercise wearing a surgical face mask (VM), and vigorous-intensity exercise not wearing a surgical face mask (VNM) (Fig. 2a). All participants visited a laboratory exercise chamber for screening and familiarization trials. After 1 week, each participant completed the six main trials at 1-week intervals, following a random order assigned using an online randomization tool [30]. All six main trials were conducted in an exercise chamber set at 30 ± 1 ℃, humidity at 70 ± 3%, and oxygen at 20.8% (Fig. 2b). These experimental conditions were designed to replicate a typically hot and humid environment, consistent with conditions set in prior research [19, 22, 24]. All the participants were requested to maintain their usual activities of daily living, except during the main trial.

### Screening and familiarization trial

All potential participants were invited to undergo screening. After signing a consent form, the participants completed health history and physical activity readiness questionnaires. Trained research assistants measured each participant’s body height (Stadiometer Seca 213, Leicester) and weight (MC-780MA, Tanita, Japan) and calculated the BMI. Subsequently, all the participants were required to complete a maximal oxygen consumption (VO_2max_) test on a treadmill using the Bruce protocol [31] to determine their maximum heart rate (HR_max_), which was assessed using Polar HR telemetry (H10 Sensor, Polar, Finland). The corresponding speed for different intensity levels (light, moderate, and vigorous) was determined for each participant, and the participants were permitted to have a short familiarization session in the chamber.

### Main trial

Each participant underwent six trials at three different exercise intensities performed either with or without a surgical face mask, with each trial lasting 15 min to ensure standardization in duration across all conditions and to address safety concerns. The 15-min duration was selected as it represents a reasonably challenging yet safe exercise timeframe for the general public, particularly during vigorous-intensity exercise while wearing a surgical face mask. The LM and LNM trials involved walking at 50% of HRmax, the MM and MNM trials involved brisk walking at 65% of HR_max_, and the VM and VNM trials involved jogging/running at 80% of HR_max_. Standard disposable three-layer surgical face masks with ear loops (Winner Medical Co., Ltd., Shenzhen, China) were used. Each face mask was used only once. No water consumption was permitted during the trial. Several outcomes were continuously monitored during exercise, including the rating of perceived exertion (RPE), oxygen saturation (SpO_2_), forehead temperature, core temperature, HR, and respiratory parameters. A certified nurse collected venous blood samples in ethylenediaminetetraacetic acid (EDTA)-coated tubes before and immediately after each trial. Blood pressure (BP) and lactate levels were measured after blood sample collection. FMD was measured before and after each trial. All post-exercise physiological measurements were completed within 15–30 min following the end of exercise. The participants completed a set of questionnaires at the conclusion of the experimental trials.

### Measurements

#### Primary outcome

##### Cardiac biomarkers (cytokine)

The plasma was separated using EDTA-coated tubes and centrifuged at 3000 rpm at 4 °C for 15 min using a Thermo Scientific (Waltham, MA, USA) centrifuge [32]. The plasma was aliquoted into Eppendorf tubes and stored in a deep freezer for further analysis. NT-proBNP and TNNT1 were analyzed using ELISA kits (Thermo Scientific) according to the manufacturer's instructions and read using a microplate absorbance spectrophotometer (xMark™, BIO-RAD). CK-MB was measured using a cobas® c 111 analyzer (Roche Diagnostics, Rotkreuz, Switzerland).

#### Secondary outcome

##### Lactate

Blood lactate levels were measured using capillary blood samples taken from the fingertips with a portable analyzer (Lactate Plus, Nova Biomedical, Waltham, Massachusetts, USA) before and immediately after each trial.

##### FMD

FMD of the brachial artery was assessed in a quiet, air-conditioned room maintained at 22–25 ℃. The participants were positioned supine and rested for 15 min with a BP cuff placed around the right forearm and ECG electrodes positioned around both wrists. FMD was measured using an ultrasound system (UNEXEF38G; UNEX, Nagoya, Japan) equipped with a 7.5 MHz linear array transducer and an automated edge-tracking function, which generated longitudinal and transverse B-mode images. The brachial artery was located 5–10 cm above the elbow, and the baseline diameter (D baseline) was recorded using the edge-tracking function. The BP cuff was inflated 50 mmHg above systolic pressure for 5 min, followed by continuous recording of brachial artery diameter changes during a 3 min decompression period. The peak brachial artery diameter (D peak) was automatically tracked, and FMD was calculated as the percentage increase as follows:

1 $$\mathrm{FMD}\;(\%)=(\mathrm D\;\mathrm{peak}-\mathrm D\;\mathrm{baseline})/(\mathrm D\;\mathrm{baseline})\times100$$

All procedures were performed by a trained, professional operator using standardized protocols [33].

##### BP

BP was measured using a clinical automatic BP monitor (M7 Intelli IT, Omron, Japan), with the cuff placed around each participant’s brachial artery. Prior to exercise, all participants were required to rest for 10 min. The BP measurements were conducted immediately post-exercise with each participant seated. Two readings were obtained for each test, and the results were averaged for systolic BP (SBP) and diastolic BP (DBP).

##### Thermoregulatory measurements

Core temperature, recorded every 30 s, was measured using a silicone-coated pill (BodyCAP, France) that was swallowed approximately 4 h before each exercise session. This timing ensured that the pill would pass through the stomach and remain unaffected by any hot or cold liquids ingested, in accordance with a previous study [34]. Forehead temperature was measured every 3 min using a No-Touch Thermometer. Similarly, SpO_2_ was measured noninvasively every 3 min using a portable finger pulse oximeter (500 BL, Zacurate, Texas, USA) throughout the trials.

##### Respiratory parameters

The participants were fitted with metabolic testing equipment (MAX-II; Physio-Dyne, New York, USA) for all six trials to continuously monitor their respiratory parameters. The system was calibrated prior to testing, including flow calibration with a 3L syringe and gas calibration using precision mixtures of 16% O_2_ and 4% CO_2_, in accordance with manufacturer guidelines and established standards to ensure accurate pulmonary gas exchange measurements [35]. Spirometry masks were fitted specifically to each participant’s mouth and nose, whether wearing a surgical face mask or not during the exercise trials, and the fit was checked for leakage. Several respiratory parameters were measured, and data were recorded at 30 s intervals, including oxygen uptake (VO_2_), minute ventilation (VE), tidal volume (VT), carbon dioxide uptake (VCO_2_), the respiratory exchange ratio (RER), and breathing frequency (BF).

##### Psycho-perceptual responses

RPE was used to indicate the participants’ physical exertion using the Borg Scale (ranging from 6 to 20) [36] at 3 min intervals during all trials. We assessed each participant’s comfort or discomfort after each trial across ten domains: breathing resistance, tightness, feeling unfit, humidity, heat, odor, fatigue, itchiness, saltiness, and overall discomfort, using a visual analog scale (VAS) ranging from 0 to 10 [19]. Additionally, the perceived enjoyment of each experimental trial was assessed using the Physical Activity Enjoyment Scale (PACES). The participants completed this 7-point bipolar scale after each trial to determine which exercise protocol was perceived as more enjoyable [37]. Furthermore, the Chinese Self-Efficacy for Exercise Scale (SEE-C) [38] was used to examine the participants’ feelings of competence and confidence during each trial.

### Statistical analysis

The descriptive statistics were summarized and reported as means ± standard deviation for continuous variables and as proportions for categorical variables. Normal distribution of the data was visually assessed using QQ plots and skewness analysis. An independent *t*-test was used to examine differences between baseline characteristics of the male and female participants. Data were analyzed using a generalized estimating equation (GEE) with Bonferroni post hoc comparisons, including factors such as surgical face mask wearing, exercise intensity, time, and their interactions in relation to outcomes measured before and after each trial. GEE was selected to account for within-subject correlations across the six trials, accommodate potential non-normal distributions (e.g., skewed biomarker data), and handle missing data robustly, unlike repeated-measures ANOVA, which assumes normality and sphericity [39]. Continuous data collected during the exercise (RPE, forehead temperature, HR, core temperature, SpO_2_, and respiratory parameters) were converted into mean values. GEE was also used to examine differences among mean values across the six trials, including factors such as mask wearing and exercise intensity. The *p*-value threshold was set at *p* < 0.05. Data were analyzed using SPSS version 27.0 software.

## Results

Of 184 potential participants, 36 were excluded after the screening test for the following reasons: BMI, > 25 kg/m^2^ (*n* = 21); the presence of a chronic disease or medication use (*n* = 10); and sports injuries (*n* = 5). In addition, 42 participants declined to participate mainly owing to insufficient time available for participation. Finally, 106 individuals participated in the main trial, and 98 completed all six exercises with 8 participants dropping out during the trial. The participant characteristics are shown in Table 1. All participants were young adults (mean age, 20.4 years; women, *n* = 29). In general, the men had higher VO_2max_ and HR_max_ levels and thus achieved higher speeds during low, moderate, and vigorous exercise trials.

**Table 1:** Participants characteristics

Variables	Male (*n* = 69)	Female (*n* = 29)
Age (year)	20.1 ± 1.9	21.0 ± 2.1
Height (cm)	174.8 ± 5.4	163.1 ± 4.8^*^
Weight (kg)	67.2 ± 8.0	53.3 ± 5.8^*^
BMI (kg/m^2^)	21.9 ± 1.8	20.0 ± 1.7^*^
VO_2max_(ml/min/kg)	46.2 ± 7.7	35.1 ± 6.1^*^
HR_max_ (bpm)	188.3 ± 9.8	176.6 ± 14.5^*^
Speed at light intensity (km/h)	5.4 ± 0.7	4.2 ± 0.7^*^
Speed at moderate intensity (km/h)	7.3 ± 0.7	5.9 ± 0.6^*^
Speed at vigorous intensity (km/h)	9.2 ± 1.0	7.4 ± 0.7^*^

*BMI* Body mass index, *HRmax* Maximum heart rate, *VO*_*2max*_ Maximal oxygen consumption, *significant difference between male and female

As shown in Table 2, the three heart failure-related markers were examined. GEE analysis revealed a significant time effect on CK-MB (*p* < 0.001), NT-proBNP (*p* < 0.001), and TNNT1 (*p* < 0.001) levels, and an intensity effect on CK-MB (*p* = 0.04) levels. After exercise, the CK-MB level was higher in the VM than in the LM trial (3.06 ng/mL, 95% confidence interval [CI]: 0.37–5.76 ng/mL, *p* = 0.013) and in the VNM than in the LM (3.52 ng/mL, 95% CI: 0.55–6.50 ng/mL, *p* = 0.008). Three trials showed higher levels of CK-MB after exercise than before exercise, including MM (2.04 ng/mL, 95% CI: 0.77–3.32 ng/mL, *p* < 0.001), VM (3.85 ng/mL, 95% CI: 2.40–5.29 ng/mL, *p* < 0.001), and VNM (3.57 ng/mL, 95% CI: 2.19–4.95 ng/mL, *p* < 0.001). In addition, three trials observed higher NT-proBNP levels than the pre-exercise levels, including VM (0.082 ng/mL, 95% CI: 0.04–0.10 ng/mL, *p* < 0.001), MNM (0.05 ng/mL, 95% CI: 0.01–0.08 ng/mL, *p* = 0.004), and VNM (0.06 ng/mL, 95% CI: 0.02–0.09 ng/mL, *p* < 0.001). Similarly, TNNT1 was higher in most trials after exercise, including the LM (0.59 ng/mL, 95% CI: 0.13–1.05 ng/mL, *p* = 0.009), VM *(*0.91 ng/mL, 95% CI: 0.49–1.33 ng/mL, *p* < 0.001), LNM (0.82 ng/mL, 95% CI: 0.28–1.35 ng/mL, *p* = 0.001), MNM (0.60 ng/mL, 95% CI: 0.19–1.02 ng/mL, *p* = 0.002), and VNM (0.81 ng/mL, 95% CI: 0.27–1.36 ng/mL, *p* = 0.001) trials. Both time and intensity had significant effects (*p* < 0.001) on the FMD, a marker of endothelial function. Specifically, a higher FMD value was observed in higher intensity trials after 15 min of exercise: VM > LM (4.11%, 95% CI: 2.96–5.26%, *p* < 0.001), MM > LM (2.39%, 95% CI: 1.11–3.68%, *p* < 0.001), VM > MM (1.72%, 95% CI: 0.35–3.09%, *p* = 0.003), VNM > LNM (3.90%, 95% CI: 2.61–5.19%, *p* < 0.001), and MNM > LNM (2.71%, 95% CI: 1.37–4.05%, *p* < 0.001). All six trials showed significantly increased FMD values after exercise.

**Table 2:** Cardiovascular-related outcomes in different trials at pre-test and post-test (*N* = 98)

		**Exercise with surgical face mask**			**Exercise without surgical face mask**			***P* value**		
**Variables**	**Time**	**Light**	**Moderate**	**Vigorous**	**Light**	**Moderate**	**Vigorous**	**Time**	**Mask**	**Intensity**
CK-MB^*^ (ng/mL)	Pre	11.5 ± 5.9	12.0 ± 6.3	11.6 ± 5.8	11.6 ± 6.8	12.5 ± 7.6	12.3 ± 7.0	< 0.001	0.52	0.04
	Post	12.4 ± 5.9	14.1 ± 7.4	15.4 ± 6.5	13.0 ± 8.8	13.9 ± 8.4	15.9 ± 7.8			
NT-proBNP^*^(ng/mL)	Pre	0.3 ± 0.2	0.3 ± 0.2	0.3 ± 0.2	0.3 ± 0.2	0.3 ± 0.2	0.3 ± 0.2	< 0.001	0.95	0.82
	Post	0.3 ± 0.2	0.3 ± 0.3	0.4 ± 0.3	0.3 ± 0.3	0.3 ± 0.2	0.4 ± 0.3			
TNNT1^*^ (ng/mL)	Pre	4.5 ± 3.2	4.7 ± 3.3	4.5 ± 3.2	4.3 ± 2.7	4.3 ± 2.9	4.2 ± 2.6	< 0.001	0.52	1.00
	Post	5.1 ± 3.5	5.2 ± 3.1	5.4 ± 3.5	5.1 ± 3.2	4.9 ± 2.8	5.1 ± 2.9			
DBP (mmHg)	Pre	67.9 ± 8.8	69.1 ± 8.2	68.1 ± 8.3	67.9 ± 8.4	70.4 ± 9.7	67.7 ± 9.1	< 0.001	0.57	< 0.001
	Post	70.9 ± 7.5	72.6 ± 7.4	69.5 ± 8.9	71.5 ± 7.6	73.3 ± 8.0	69.4 ± 8.7			
SBP (mmHg)	Pre	111.5 ± 12.6	112.4 ± 12.5	112.3 ± 13.1	112.8 ± 12.5	113.0 ± 13.0	110.5 ± 12.0	< 0.001	0.82	< 0.001
	Post	116.3 ± 12.4	121.6 ± 14.4	125.0 ± 15.0	115.1 ± 12.8	120.6 ± 14.6	125.8 ± 16.5			
FMD^#^ (%)	Pre	10.7 ± 1.7	10.8 ± 2.8	10.4 ± 2.1	10.7 ± 2.6	10.7 ± 2.2	10.6 ± 1.7	< 0.001	0.27	< 0.001
	Post	11.7 ± 2.3	14.1 ± 3.3	15.8 ± 2.8	12.0 ± 2.8	14.7 ± 3.2	15.9 ± 2.9			
Lactate (mmol/L)	Pre	1.7 ± 1.0	1.8 ± 1.1	1.9 ± 1.1	1.9 ± 1.1	1.9 ± 1.2	1.9 ± 1.1	< 0.001	0.73	< 0.001
	Post	2.5 ± 2.1	3.2 ± 1.9	5.9 ± 3.2	1.9 ± 1.7	3.3 ± 2.4	5.8 ± 3.1			

*CK-MB* Creatine kinase-MB, *DBP* Diastolic blood pressure, *FMD* Flow-mediated dilation, *NT-proBNP* N-terminal pro-brain natriuretic peptide, *SDP* Systolic blood pressure, *TNNT1* Troponin T type 1

^*^As some sample is lower than the measurement range, there were some missing data for the above sample, including CK-MB (*n* = 8); NT-proBNP (*n* = 19); TNNT1 (*n* = 45)

^#^Limitations of equipment for FMD measurement, FMD (*n* = 85)

DBP and SBP showed significant effects on exercise time and intensity (*p* < 0.001; Table 2). Specifically, DBP significantly increased in the LM (3.05 mmHg, 95% CI: 0.69–5.41 mmHg, *p* = 0.010), MM (3.52 mmHg, 95% CI: 1.33–5.71 mmHg, *p* < 0.001), and LNM (3.92 mmHg, 95% CI: 0.17–7.68 mmHg, *p* < 0.001) trials after exercise compared with baseline. Only the MNM trial showed a significantly higher DBP than the VNM after 15 min of exercise (3.92 mmHg, 95% CI: 0.17–7.68 mmHg, *p* = 0.033). After exercise, SBP was higher in the VM than in the LM (8.66 mmHg, 95% CI: 2.51–14.81 mmHg, *p* = 0.001) and higher in the VNM than in the LNM (10.63 mmHg, 95% CI: 4.01–17.25 mmHg, *p* < 0.001). Five trials showed significantly increased SBP after 15-min exercise, including the LM (4.79 mmHg, 95% CI: 1.87–7.70 mmHg, *p* < 0.001), MM (9.19 mmHg, 95% CI: 5.85–12.53 mmHg, *p* < 0.001), VM (12.67 mmHg, 95% CI: 8.33–17.01 mmHg, *p* < 0.001), MNM (7.55 mmHg, 95% CI: 3.90–11.21 mmHg, *p* < 0.001), and VNM (15.29 mmHg, 95% CI: 11.28–19.29 mmHg, *p* < 0.001). The lactate levels were significantly higher in the trials, i.e., LM (0.78 mmol/L, 95% CI: 0.23–1.33 mmol/L, *p* < 0.001), MM (1.33 mmol/L, 95% CI: 0.83–1.84 mmol/L, *p* < 0.001), VM (3.99 mmol/L, 95% CI: 3.08–4.89 mmol/L, *p* < 0.001), MNM (1.43 mmol/L, 95% CI: 0.79–2.08 mmol/L, *p* < 0.001), and VNM (3.90 mmol/L, 95% CI: 3.00–4.81 mmol/L, *p* < 0.001) after exercise. In addition, a higher lactate level was found in the higher-intensity trial compared with the lower-intensity trial: VM > LM (3.44 mmol/L, 95% CI: 2.24–4.65 mmol/L, *p* < 0.001), VM > MM (2.76 mmol/L, 95% CI: 1.59–3.92 mmol/L, *p* < 0.001), MNM > LNM (1.45 mmol/L, 95% CI: 0.52–2.38 mmol/L, *p* < 0.001), VNM > LNM (3.91 mmol/L, 95% CI: 2.80–5.02 mmol/L, *p* < 0.001), and VNM > MNM (2.45 mmol/L, 95% CI: 1.22–3.70 mmol/L, *p* < 0.001).

**Fig. 3:**
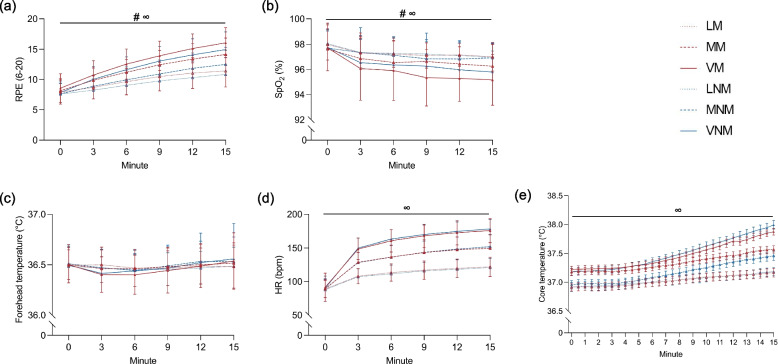
The change of physiological outcomes in different trials (**a**) RPE: rating of perceived exertion; (**b**) SPO2: oxygen saturation; (**c**) forehead temperature; (**d**) HR: heart rate; (**e**) core temperature. #: significant difference (p<0.05) between wearing a surgical face mask and not wearing a surgical face mask; ∞: significant difference (p<0.05) among the three exercise intensity levels. LM: light-intensity exercise wearing a surgical face mask; LNM: light-intensity exercise not wearing a surgical face mask; MM: moderate-intensity exercise wearing a surgical face mask; MNM: moderate-intensity exercise not wearing a surgical face mask; VM: vigorous-intensity exercise wearing a surgical face mask; VNM: vigorous-intensity exercise not wearing a surgical face mask

The mean value for each trial was calculated and compared between the six trials, and is shown in Table 3. The RPE showed a significant effect in relation to mask wearing (*p* < 0.001) and exercise intensity (*p* < 0.001) (Fig. 3a). Specifically, light and moderate exercise intensities wearing a surgical face mask showed a higher RPE than exercises without a face mask. In addition, there was an increased level of RPE with an increase in exercise intensity: VM > LM (2.91, 95% CI 2.20–3.61; *p* < 0.001), VM > MM (1.26, 95% CI 0.57–1.95; *p* < 0.001), and MM > LM (1.64, 95% CI 0.96–2.33; *p* < 0.001). GEE analysis of SpO_2_ revealed a significant mask-intensity interaction effect (*p* = 0.07) (Fig. 3b). The SpO_2_ was lower in trials with a mask only during moderate (−0.40%, 95% CI −0.74–−0.06%; *p* = 0.019) and vigorous (−0.51%, 95% CI −0.92–−0.10%; *p* = 0.015) intensity exercises. In addition, the average SpO_2_ was lower in higher intensity trials: VM < LM (−1.34%, 95% CI −1.76–−0.92%; *p* < 0.001), MM < LM (−0.57%, 95% CI −0.94–−0.20%; *p* = 0.001), VM < MM (−0.78%, 95% CI −1.27–−0.28%; *p* = 0.001), and VNM < LNM (−0.87%, 95% CI −1.26–−0.48%; *p* < 0.001). For both forehead and core temperatures (Fig. 3c, e), only the intensity of core temperature showed a significant effect (*p* < 0.001): VM > LM (0.43 ℃, 95% CI 0.21–0.64 ℃; *p* < 0.001), MM > LM (0.30 ℃, 95% CI 0.08–0.51 ℃; *p* = 0.003), VNM > LNM (0.46 ℃, 95% CI 0.23–0.69 ℃; *p* < 0.001), VNM > MNM (0.33 ℃, 95% CI 0.11–0.55 ℃; *p* = 0.001). Only significant intensity effect was observed on HR (*p* < 0.001) was observed, including VM > LM (39.75 bpm, 95% CI 35.09–44.4 bpm; *p* < 0.001), VM > MM (18.86 bpm, 95% CI 13.69–24.03 bpm; *p* < 0.001), MM > LM (20.89 bpm, 95% CI 16.32–25.47 bpm; *p* < 0.001) (Fig. 3d).

**Table 3:** Physiological outcomes in different trials (*N* = 98)

Variables	Exercise with surgical face mask			Exercise without surgical face mask			*P* value		
	Light	Moderate	Vigorous	Light	Moderate	Vigorous	Mask	Intensity	Mask*Intensity
RPE	9.9 ± 2.0	11.5 ± 2.0	12.8 ± 2.1	9.3 ± 2.1	10.3 ± 2.0	11.9 ± 2.0	< 0.001	< 0.001	0.27
SpO_2_ (%)	97.3 ± 0.8	96.7 ± 1.3	95.9 ± 1.6	97.3 ± 0.9	97.1 ± 1.1	96.5 ± 1.4	0.001	< 0.001	0.07
Heart rate (bpm)	111.6 ± 11.7	132.5 ± 15.0	151.3 ± 15.4	110.3 ± 11.4	132.9 ± 14.1	154.1 ± 12.0	0.57	< 0.001	0.29
Forehead temperature (℃)	36.5 ± 0.1	36.5 ± 0.2	36.5 ± 0.1	36.5 ± 0.1	36.5 ± 0.2	36.5 ± 0.2	0.65	0.39	0.51
Core-temperature (℃)	37.0 ± 0.3	37.3 ± 0.3	37.5 ± 0.2	37.0 ± 0.3	37.2 ± 0.3	37.5 ± 0.3	0.46	< 0.001	0.24
VO_2_ (ml/min/kg)	12.9 ± 3.9	17.8 ± 5.7	22.8 ± 6.6	14.7 ± 3.7	20.9 ± 5.1	27.6 ± 6.2	< 0.001	< 0.001	0.16
VCO_2_ (ml/min/kg)	13.5 ± 3.6	19.1 ± 5.7	25.1 ± 6.6	14.8 ± 3.6	23.0 ± 5.6	32.2 ± 6.2	< 0.001	< 0.001	< 0.001
RER	1.1 ± 0.2	1.1 ± 0.2	1.1 ± 0.2	1.0 ± 0.2	1.1 ± 0.2	1.2 ± 0.2	0.47	< 0.001	0.02
VE (L/min)	24.8 ± 6.8	34.9 ± 10.9	46.7 ± 12.9	29.2 ± 7.3	43.6 ± 12.3	62.8 ± 15.5	< 0.001	< 0.001	< 0.001
VT (L)	1.0 ± 0.3	1.2 ± 0.3	1.4 ± 0.3	1.1 ± 0.3	1.4 ± 0.4	1.6 ± 0.3	< 0.001	< 0.001	0.01
BF (n/min)	24.4 ± 4.4	28.5 ± 5.5	33.9 ± 6.6	27.2 ± 4.7	31.9 ± 5.5	38.4 ± 6.3	< 0.001	< 0.001	0.35

*BF* Breathing frequency, *RER* Respiratory exchange ratio, *RPE* Rating of perceived exertion, *SpO*_*2*_ Oxygen saturation, *VCO*_*2*_ Carbon dioxide uptake, *VE* Minute ventilation, *VO*_*2*_ Oxygen uptake, *VT* Tidal volume

Several respiratory parameters were continuously monitored during the trials (Table 3). Most outcomes showed similar changes, which reached significance in the mask × intensity interaction, including VCO_2_ (*p* < 0.001), the RER (*p* = 0.02), VE (*p* < 0.001), and VT (*p* = 0.01), while only significant mask (*p* < 0.001) and exercise intensity (*p* < 0.001) individual effects on breathing frequency and VO_2_ were observed. Generally, exercise without wearing a surgical face mask at any intensity showed a higher value, and a higher value was observed in trials with a higher intensity with or without a mask: VM > MM, VM > LM, and MM > LM. However, for the RER, significant differences among the various intensities were only observed in trials without the mask.

Additionally, several psychological outcomes were assessed after each trial (Table 4). No significant effects (mask wearing, exercise intensity, and mask × intensity) were observed for either the PACES or SEEC. Additionally, several items were assessed based on the perceived discomfort level. Most items showed a significant intensity effect (*p* < 0.001), including humidity, heat, breath resistance, tightness, saltiness, lack of fitness, odor, fatigue, and overall discomfort. Four items (breath resistance, lack of fitness, fatigue, and overall discomfort) showed a significant mask effect (*p* < 0.001), although no significant mask × intensity interaction was observed for any item. In summary, exercise with a mask compared with exercise without a mask, or exercise at a higher intensity compared with a lower intensity, showed a higher level of perceived discomfort.

**Table 4:** Psychological outcomes in different trials at post-test (*N* = 98)

Variables	Exercise with surgical face mask			Exercise without surgical face mask			*P *value		
	Light	Moderate	Vigorous	Light	Moderate	Vigorous	Mask	Intensity	Mask*Intensity
PACES	81.0 ± 12.5	78.8 ± 13.2	79.6 ± 11.6	79.1 ± 12.7	81.1 ± 13.2	79.7 ± 12.4	0.86	0.93	0.26
SEEC	4.4 ± 1.4	4.5 ± 1.5	4.4 ± 1.3	4.3 ± 1.4	4.4 ± 1.4	4.4 ± 1.5	0.78	0.92	0.92
Perceived discomfort level (out of 10)									
Humid	4.5 ± 2.3	5.6 ± 2.2	6.1 ± 2.5	4.5 ± 2.3	5.3 ± 2.2	6.0 ± 2.3	0.57	< 0.001	0.89
Hot	5.3 ± 2.0	6.0 ± 1.9	6.5 ± 2.1	5.0 ± 2.2	5.8 ± 2.0	6.5 ± 1.8	0.32	< 0.001	0.75
Breath resistance	5.5 ± 2.2	7.0 ± 2.0	7.7 ± 1.9	3.7 ± 2.2	4.7 ± 2.0	5.2 ± 2.1	< 0.001	< 0.001	0.28
Itchy	1.9 ± 1.9	2.0 ± 2.0	2.0 ± 1.7	2.0 ± 2.0	1.8 ± 1.7	2.0 ± 1.9	0.95	0.95	0.62
Tightness	3.4 ± 2.2	4.0 ± 2.6	4.2 ± 2.5	2.8 ± 2.2	3.5 ± 2.0	4.0 ± 2.3	0.02	< 0.001	0.83
Salty	2.1 ± 2.0	2.8 ± 2.4	3.2 ± 2.5	1.9 ± 2.0	2.8 ± 2.2	3.2 ± 2.5	0.77	< 0.001	0.91
Unfit	3.4 ± 2.4	4.5 ± 2.6	5.5 ± 2.5	2.7 ± 2.1	3.7 ± 2.2	4.7 ± 2.5	< 0.001	< 0.001	0.96
Odor	2.0 ± 1.9	2.3 ± 2.1	2.5 ± 2.1	1.8 ± 1.8	2.3 ± 2.0	2.4 ± 2.3	0.58	0.02	0.90
Fatigue	3.4 ± 2.0	5.1 ± 2.1	6.3 ± 2.0	3.3 ± 2.1	4.5 ± 1.9	5.7 ± 2.1	< 0.001	< 0.001	0.29
Overall	3.3 ± 1.6	4.4 ± 2.1	5.4 ± 2.3	2.9 ± 1.7	3.5 ± 1.6	4.5 ± 2.0	< 0.001	< 0.001	0.37

*PACES* Physical activity enjoyment scale, *SEEC* Chinese version of the self-efficacy for exercise

## Discussion

To our knowledge, this is the first study to examine the effects of exercise while wearing a surgical face mask at different intensities under hot and humid conditions. We included 98 participants (both men and women) for six separate randomized crossover trials. The key findings of this study were as follows: (i) most physiological outcomes and biomarkers showed significant time and intensity effects at higher levels post-exercise or at higher intensity trials, despite no significant effects of surgical face mask use; (ii) most of the respiratory parameters showed a mask and intensity effect, with higher exercise intensity showing higher AUCs for respiratory parameters, while exercise wearing a surgical face mask showed lower values; and (iv) exercise while wearing a surgical face mask or exercise at higher intensity showed higher levels of perceived discomfort.

The incorporation of exploratory cardiac biomarkers was one of the strengths of this study; however, most of the markers only showed a time effect, particularly after moderate- and vigorous-intensity exercises. Similar to a previous study, all three secondary outcome levels (for CK-MB, NT-proBNP, and TNNT1) significantly increased after acute exercise [28, 40, 41] although the exercise protocol differed between that study and our study, with no difference observed between the various exercise intensities. However, no intensity or mask effect was observed in relation to NT-proBNP and TNNT1. Similarly, previous research has shown that NT-proBNP and TNNT1 elevation are primarily duration-dependent rather than intensity-dependent, with studies showing peak post-exercise concentrations significantly associated with exercise duration but not with exercise intensity [40, 41]. In addition, values for some of the participants’ samples were not detectable when following manufacturer’s guidelines, which referred to values < 0.137 ng/mL for NT-proBNP and < 0.391 ng/mL for TNNT1. Moreover, considering these markers are usually elevated 24 h post-exercise [42], we only assessed these three markers immediately post-exercise owing to practical constraints, which may not accurately reflect differences among the different trials [29, 43]. Specifically, TNNT1 peaks 2–5 h post-exercise, whereas our immediate post-exercise sampling captured only the initial phase of biomarker kinetics [41]. Caution is needed when considering the intensity effect in relation to CK-MB, as CK-MB is released by both skeletal and heart muscle, while NT-proBNP and TNNT1 are released by heart muscle only [44]. Furthermore, these elevated biomarker levels do not necessarily indicate myocardial injury, as they may reflect physiological adaptations to exercise [28]. Therefore, future studies should incorporate electrocardiograms and extended monitoring (24–48 h) to evaluate potential myocardial injury risk from such exercise protocols.

Several physiological outcomes were measured pre- and post-exercise, including DBP, SBP, and FMD. As the BP was measured immediately post-exercise, DBP and SBP increased, which is a normal hemodynamic response [45]. Specifically, SBP increased more with the increase in exercise intensity, while DBP fell slightly in all trials, and all BP values were lower than criteria for discontinuation of exercise [45]. FMD, the measurement for endothelial function, also showed time and intensity-related effects. After a high-intensity 15-min exercise, FMD increased; in contrast, some studies have reported that FMD decreases immediately post-exercise [46, 47]. Considering that several physiological measurements needed to be conducted, the real-time assessment of FMD in our study was performed approximately 15–30 min post-exercise completion, and we also required participants to rest for 15 min prior to the FMD test, which may have allowed for potential supercompensation in vascular function [48]. Previous studies have shown that FMD can increase within 2 h after high-intensity exercise, although immediate post-exercise measurements often reveal an initial decrease. Furthermore, the high-intensity protocol in this study was defined as 80% HRmax, which differs from that used in previous research (80% VO_2peak_) [48]. However, no mask effect was found, which may have been because the 15-min exercise period was not sufficiently long to result in vascular-related changes.

Most of the respiratory parameters showed differences between trials with or without surgical face masks. VO_2_, VCO_2_, VE, VT, and breathing frequency values were lower in exercise trials when wearing a surgical face mask. These reductions align with those of Lassing et al. [49], who reported decreased VE, VO_2_, and breathing frequency owing to increased airway resistance. Conversely, Braga et al. reported a small increment in VO_2_ and VCO_2_ values [50]. In a meta-analysis by Shaw et al. [51] reduced VE was reported; however, their result contrasts with our findings concerning reduced SpO2, VT, and breathing frequency, suggesting variability from compensatory breathing patterns (e.g., slower, deeper breaths) or unnoticed leaks in pulmonary gas testing, as the spirometry mask over the surgical mask may have compromised seal integrity [51]. Future studies should standardize mask fit and enhance leak detection to address these inconsistencies.

Moreover, our participants reported a higher level of perceived discomfort regarding breath resistance, lack of fitness, fatigue, and overall scores when wearing a surgical mask during exercise. Our discomfort questionnaire has also been used in previous studies involving wearing a cloth mask for maximal cardiopulmonary exercise tests [13], wearing a surgical mask for an 18-min incremental treadmill exercise [28], and wearing cloth or surgical masks for a 1-min sit-to-stand test [52]. All the above-mentioned studies focused on general health, with the scores being comparable among the studies, ranging from 4 to 6 for the overall discomfort level, which is indicative of mild discomfort. However, we consider our findings to be reasonable, as the subjective measurements were subject to the participants’ understanding and the researchers’ interpretation of the questionnaire. However, the highest scores were observed under hot and humid conditions, apart from breath resistance, among all the sub-items in this questionnaire, which reflected the participants’ subjective feelings for this condition. In relation to the SEEC, no significant effects were observed. While the SEEC is designed to facilitate participants’ confidence in continuing exercise when meeting some barriers, the questions involved in this questionnaire may be more suitable for long-term intervention, as shown in a higher SEEC score observed after a 3-month home exercise program during the COVID-19 pandemic [38]. This may explain why no significant effect was observed using the SEEC after six trials in a well-controlled experimental laboratory [53]. Similarly, no effect was identified using the PACES, which is consistent with a previous study that reported enjoyment did not vary among various high-intensity interval exercises [54].

This study had several strengths. We included a relatively large sample size of 98 participants and utilized a randomized crossover trial design, which ensured a robust methodological approach. All the trials were conducted in a temperature- and humidity-controlled chamber to strengthen the data quality. Various physiological effects were assessed during the trials, and venous blood samples were collected for cardiac arrest-related biomarkers, which had not been undertaken to the same extent in previous studies. However, our study also had some limitations. Measuring BP immediately post-exercise, rather than continuously during exercise, may not fully capture intra-exercise hemodynamic responses, potentially introducing systematic bias owing to rapid BP recovery post-exercise. Future studies should consider including continuous BP monitoring during exercise to improve the accuracy of hemodynamic data. Additionally, the 15-min trial duration, selected for safety and to standardize exercise time across the trials, may have limited the detection of significant differences in some outcomes and the translation of findings to real-world settings, where individuals typically exercise for > 15 min per session. However, this protocol duration remained challenging for some participants, particularly at vigorous intensity. Only immediate post-exercise cardiac biomarker levels (i.e., TNNT1, NT-proBNP, CK-MB) were measured owing to practical constraints, such as participant burden and safety concerns in hot, humid conditions, unlike studies tracking biomarker kinetics > 24 h that require extensive monitoring. Similarly, FMD was measured 15–30 min post-exercise, a window where a transient decrease is typically expected, and timing was not standardized across the participants because of practical constraints, potentially affecting result consistency. Moreover, the placement of a spirometry mask over the surgical face mask, necessary for gas exchange measurements [13, 14, 49], may have amplified perceptual discomfort and breathing resistance in the high-humidity environment, potentially exacerbating carbon dioxide rebreathing [25]. All of the study participants were healthy and generally young; hence, our results may not be generalizable to other populations, particularly to patients with heart disease, who may more easily experience adverse cardiovascular adaptations during high-intensity training [55]. Furthermore, the habitual physical activity levels of the participants were not assessed, which may influence physiological responses to exercise. Future studies are required that consider including physical activity assessments to investigate its potential moderating effects.

## Conclusion

Wearing a surgical face mask during exercise in hot, humid conditions had no significant effect on most cardio-related parameters, which were primarily influenced by exercise intensity and time. However, surgical face mask use had a significantly impairing effect in relation to the respiratory parameters, and increased perceived discomfort compared with exercising without a surgical face mask. These findings suggest that while surgical face masks do not appear to exacerbate cardiovascular stress during exercise in hot, humid environments, their effect on respiratory function and comfort warrants consideration for prolonged or high-intensity activities.

## Supplementary Information

Supplementary Material 1.

## Data Availability

Data generated or analyzed during this study are available from the corresponding author upon reasonable request.
