# Supplementary material for: Effects of wearing a surgical face mask on cardiac biomarkers, respiratory function, and perceptual responses during exercise in a hot and humid climate at different intensities: a randomized crossover trial
Source: BMC Sports Sci Med Rehabil. 2026 Feb 3;18:108. doi: 10.1186/s13102-026-01532-z (PMC12964896; doi:10.1186/s13102-026-01532-z)
Supplement: Supplementary file 1 — Supplementary Material 1. [file 13102_2026_1532_MOESM1_ESM.docx]

**CONSORT checklist**

| Item | Description | Page No |
| --- | --- | --- |
| 1a | Identification as a randomised crossover trial in the title | 1 |
| 1b | Specify a crossover design and report all information outlined in table 2 | 2 |
| 2a | Scientific background and explanation of rationale | 3-4 |
| 2b | Specific objectives or hypotheses | 4 |
| 3a | Rationale for a crossover design. Description of the design features including allocation ratio, especially the number and duration of periods, duration of washout period, and consideration of carry over effect | 4-5 |
| 3b | Important changes to methods after trial commencement (such as eligibility criteria), with reasons | / |
| 4a | Eligibility criteria for participants | 5 |
| 4b | Settings and locations where the data were collected | 4-5 |
| 5 | The interventions with sufficient details to allow replication, including how and when they were actually administered | 5 |
| 6a | Completely defined prespecified primary and secondary outcome measures, including how and when they were assessed | 6 |
| 6b | Any changes to trial outcomes after the trial commenced, with reasons | 6 |
| 7a | How sample size was determined, accounting for within participant variability | 4 |
| 7b | When applicable, explanation of any interim analyses and stopping guidelines | / |
| 8a | Method used to generate the random allocation sequence | 5 |
| 8b | Type of randomisation; details of any restriction (such as blocking and block size) | 5 |
| 9 | Mechanism used to implement the random allocation sequence (such as sequentially numbered containers), describing any steps taken to conceal the sequence until interventions were assigned | 5 |
| 10 | Who generated the random allocation sequence, who enrolled participants, and who assigned participants to the sequence of interventions | 5 |
| 11a | If done, who was blinded after assignment to interventions (for example, participants, care providers, those assessing outcomes) and how | 5 |
| 11b | If relevant, description of the similarity of interventions | / |
| 12a | Statistical methods used to compare groups for primary and secondary outcomes which are appropriate for crossover design (that is, based on within participant comparison) | 7 |
| 12b | Methods for additional analyses, such as subgroup analyses and adjusted analyses | / |
| 13a | The numbers of participants who were randomly assigned, received intended treatment, and were analysed for the primary outcome, separately for each sequence and period | 7 |
| 13b | No of participants excluded at each stage, with reasons, separately for each sequence and period | 7 |
| 14a | Dates defining the periods of recruitment and follow-up | 4 |
| 14b | Why the trial ended or was stopped | 7 |
| 15 | A table showing baseline demographic and clinical characteristics by sequence and period | Table 1 |
| 16 | Number of participants (denominator) included in each analysis and whether the analysis was by original assigned groups | 7 |
| 17a | For each primary and secondary outcome, results including estimated effect size and its precision (such as 95% confidence interval) should be based on within participant comparisons. In addition, results for each intervention in each period are recommended | 8-9 |
| 17b | For binary outcomes, presentation of both absolute and relative effect sizes is recommended | / |
| 18 | Results of any other analyses performed, including subgroup analyses and adjusted analyses, distinguishing prespecified from exploratory | / |
| 19 | Describe all important harms or untended effects in a way that accounts for the design (for specific guidance, see CONSORT for harms) | / |
| 20 | Trial limitations, addressing sources of potential bias, imprecision, and if relevant, multiplicity of analyses. Consider potential carry over effects | 11 |
| 21 | Generalisability (external validity, applicability) of the trial findings | 11 |
| 22 | Interpretation consistent with results, balancing benefits and harms, and considering other relevant evidence | 9-11 |
| 23 | Registration number and name of trial registry | 2 |
| 24 | Where the full trial protocol can be accessed, if available | / |
| 25 | Sources of funding and other support (such as supply of drugs), role of funders | 12 |
